# Automated data extraction tool (DET) for external applications in radiotherapy

**DOI:** 10.1016/j.tipsro.2022.12.001

**Published:** 2022-12-20

**Authors:** Mruga Gurjar, Jesper Lindberg, Thomas Björk-Eriksson, Caroline Olsson

**Affiliations:** a Medical Radiation Sciences, Institute of Clinical Sciences, Sahlgrenska Academy, University of Gothenburg, Sweden; b Department of Medical Physics and Biomedical Engineering, Sahlgrenska University Hospital, Gothenburg, Sweden; c Regional Cancer Centre West, Western Sweden Healthcare Region, Gothenburg, Sweden; d Department of Oncology, Institute of Clinical Sciences, Sahlgrenska Academy, University of Gothenburg, Sweden

**Keywords:** Radiotherapy, Data extraction, Data cleaning, Automation

## Abstract

Oncological Information Systems (OIS) manage information in radiotherapy (RT) departments. Due to database structure limitations, stored information can rarely be directly used except for vendor-specific purposes. Our aim is to enable the use of such data in various external applications by creating a tool for automatic data extraction, cleaning and formatting. Methods and materials: We used OIS data from a nine-linac RT department in Sweden (70 weeks, 2015–16). Extracted data included patients’ referrals and appointments with details for RT sub-tasks. The data extraction tool to prepare the data for external use was built in C# programming language. It used excel-automation queries to remove unassigned/duplicated values, substitute missing data and perform application-specific calculations. Descriptive statistics were used to verify the output with the manually prepared dataset from the corresponding time period. Results: From the initial raw data, 2030 (51 %)/907 (23 %) patients had known curative and palliative treatment intent for 84 different cancer diagnoses. After removal of incomplete entries, 373 (10 %) patients had unknown treatment intents which were substituted based on the known curative/palliative ratio. Automatically- and manuallyprepared datasets differed < 1 % for Mould, Treatment planning, Quality assurance and ± 5 % for Fractions and Magnetic resonance imaging with overestimations in 80/140 (57 %) entries by the tool. Conclusion: We successfully implemented a software tool to prepare ready-to-use OIS datasets for external applications. Our evaluations showed overall results close to the manually-prepared dataset. The time taken to prepare the dataset using our automated strategy can reduce the time for manual preparation from weeks to seconds.

## Highlights

• DET generates ready-to-use reliable datasets and statistics from radiotherapy oncology information systems.
• The user-friendly GUI supports easy extraction of information for external use in clinical or non-clinical settings.
• Automatic data extraction and cleaning ensure fast data processing and reduce manual processing time.

## Introduction

Oncology Information Systems (OISs) are used to manage information in radiotherapy (RT) departments. With the vast inflow of cancer patients, large amounts of treatment-related data are continuously added to the OIS, partly also including information on patient demographics. These OIS data are valuable to assist in answering clinical as well as research questions and to provide quality insights for organizational improvements of RT. However, due to a lack of guidelines for data entry and database limitations in OISs, stored information can rarely be directly used for other than vendor-specific purposes [1], [2]. To enable use in various external applications, the raw data stored in OISs first need to be cleaned and formatted to fit their required input formats.

Use of external applications with comprehensive high quality and consistent datasets is an important step to broaden the usage of OIS data from RT departments [3]. Examples of such applications include decision-support tools where RT staff can be assisted in decisions on resource allocations according to available capacity [4], [5]. Other examples concern usage of dose/volume metrics in clinical studies, for quality assurance purposes or for dose–response modelling [6], [7]. Regardless of application, preparing OIS data for external use can be a lengthy (manual) task, particularly for situations where new datasets need to be extracted frequently [2], [5]. Data efforts in RT rarely focus on the practical side of data collection and extraction as most of the research focuses on development of applications to improve the workflow or other aspects of RT [8]. So far, there are only a few contributors who emphasize the importance of creating means such as automated strategies for data retrieval to enable increased use of RT data outside OISs [2].

In this study, we describe practical aspects of automating the process of preparing OIS data for external use. The purpose of this work is to create and verify a data extraction tool for a commercial OIS, to automate extraction, cleaning, and formatting of data for use in external applications. We tested the data extracted from the tool as input to an example external application to confirm and update previously reported results on resource use from a simulation model over the RT process [4].

## Materials and methods

### Data characteristics

Information used for development and testing of the data extraction tool in this study was collected from a nine-linac RT department in Sweden. The OIS ARIA (Varian Medical Systems, Inc., Palo Alto, CA, U.S.A.) used in the department includes information about patient demographics, pre-treatment tasks including imaging, quality assurance (QA), treatment planning, and treatment delivery.

Information required from the OIS ARIA for the example external application used to test performance of the tool (described below), needed to include specifics about each patient’s treatment path, divided into three parts. One part, **referral data**, with diagnosis code (ICD-10), treatment intent, and referral start dates. Another part, **appointment data**, with appointment details for mould, imaging (computed tomography [CT], magnetic resonance imaging [MRI], positron-emission tomography [PET]), and QA with unique appointment identifiers (ids). The third part, **fraction data**, included number of fractions corresponding to the appointment data.

For tool development and verification, we used extracted data for a 16-month period (January 2015-April 2016) which were manually cleaned and formatted, referred to as the reference dataset. Subsequently, all the manual steps taken to prepare the data for the example external application were automated in the tool. The investigated application also required the structuring of data according to the largest diagnosis and treatment intent groups (corresponding to 80 % of data) and associated statistics for usage of tasks/resources in the RT workflow.

Extracted raw data from OIS ARIA included inconsistencies in variables for both referral and appointment data, which had to be cleaned to meet the associated input data requirements. The raw data also included missing information which was handled by applying different substitution strategies per diagnosis (all assigned to 1. curative, 2. palliative, 3. equal split between curative/palliative, or 4. known ratio of curative/palliative; details in supplementary material).

### Tool development

**Fig. 1:**
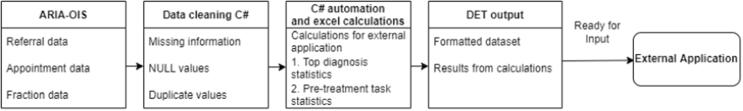
Data Extraction Tool (DET) process. Stepwise automation of extraction, cleaning and formatting of data from the ARIA oncology information system (OIS) to fit the input data format of the here investigated example external application. Details of calculation statistics are given in the results.

**Fig. 2:**
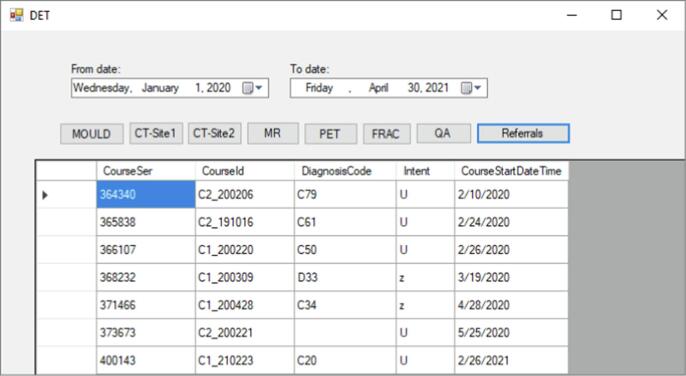
Extraction tool graphical user interface. Dates are selected using the date picker at the top of the window. Clicking Referrals extracts all referral data for the selected time period presented in the white boxes. Similarly, selecting a sub-task will result in an extraction of all appointment data related to the sub-task. Abbreviations: CT = computed tomography, CT-site1 = CT at main department, CT-site2 = CT at satellite department, DET = Data Extraction Tool, MR = magnetic resonance imaging, PET = positron emission tomography, FRAC = fractions, QA = quality assurance.

Details on the development of the tool can be found in the supplementary material. In short, the data extraction tool, primarily built in C# (Visual Studio IDE, Version 17.1, Microsoft, Washington, U.S.A), included excel-automation queries to remove unassigned/duplicated values, substitution of missing data, and execution of application-specific calculations. The overall process from retrieval of raw OIS data to cleaned and formatted input data ready for external use is presented in Fig. 1 and in Table 1. The cleaning and formatting steps take place on the back-end but are triggered by the user via the tool’s graphical user interface (GUI; Fig. 2).

**Table 1:** The synchronization between front-end and back-end operations of DET to automatically extract and export datasets for use in external applications, developed with C# in Microsoft’s Visual Studio Application.

**Process**	**Front End**	**Back End**
**1.**	**Start** application Specify time-period	1. Server connectivity established
**2.**	**Select** Referral/Appointment subtask/Fraction data (Click Event)	1. Database connectivity established Raw data extracted based on the specified time period and saved in an internal file (non-accessible for user) at a pre-defined location 2. Existing Excel file with automated formulae for data cleaning automatically linked to the extracted file 3. Cleaned data displayed on the GUI
**3.**	**Select** Export/Save to a user-defined location (Click Event)	1. Cleaned data exported and existing Excel file with automated formulae for statistics (specific to the external application in use) automatically linked to the exported/saved file (user-accessible file 1) 2. Statistics exported on application-specific format to a new excel file (user-accessible file 2)
4.	**Loop** according to default time interval settings or to user-defined time intervals	

Abbreviation: GUI: Graphical User interface.

### Tool verification

#### Comparison of manually cleaned data vs automatically cleaned data

We compared the reference dataset with the automatically-cleaned dataset for the same time period of 2015–16 (tool dataset). We considered three comparison points for verification purposes. For the data cleaning and formatting steps, we compared the ratio of curative to palliative referrals. The ratio was different for each diagnosis; hence, diagnose-specific ratios were assessed. We also compared the diagnosis-intent groups found after manual calculations and after automated calculations from the tool. Finally, the percentage of null values were removed, and percentage of duplicates removed for referral and appointment data were assessed for the tool dataset.

### External application example

#### A simulation model of the RT workflow

The simulation model used to evaluate the data from the extraction tool illustrates resource utilization at the same RT department as the data used for tool development were taken from. The intended use of the model was to help the department to plan for resource allocations [4]. The model separates the RT preparatory part from the treatment part and has previously been used to investigate different scenarios around the Swedish summer vacation period (June-August). Using the abovementioned reference dataset from 2015 to 2016, the most preferable scenario, minimizing the impact on the overall patient throughput without violating legislated staff vacation rights, was identified when the preparatory part vacation period started 1–2 weeks prior to the treatment part vacation period. To test performance of the extraction tool in practice, the original simulation results based on the reference dataset were visually compared to simulation results based on the tool dataset with respect to patterns of patients waiting for preparations, patients waiting for treatment, and patients under treatment. An automatically formatted second dataset for a comparable, but more recent, time period was also investigated (January 2020 to April 2021).

## Results

### Data characteristics – Automatically cleaned data from 2015 to 2016

For the same time period as for the reference dataset, the raw data behind the tool dataset included 3916 patients before being automatically cleaned. The number of patients treated with known curative intent was 2030 (51 %) and with known palliative intent was 907 (23 %). Number of appointments ranged between 684 (MR) to 3735 (CT) with 58,228 fractions in total for all patients.

### Removal of duplicates and null values

For referral data, after removal of duplicates and null values, the total number of patients was 3310 (Table 2). Patient referral data had a lower number of duplicates (1 %) and higher number of NULL diagnose entries (15 %) in comparison with all sub-tasks from appointment data where number of duplicates was higher (2–24 %) and number of NULL entries was lower (1–4 %). For appointment data, MR had the lowest number for both duplicates and NULL (2 %), QA had the highest number of duplicates (24 %), and Mould had the highest number of NULL (4 %). Patient fraction data had ≤ 1 % duplicates and ≤ 1 % NULL entries.

**Table 2:** Data-cleaning statistics for the automatically-cleaned dataset from 2015 to 16 including original (raw data) values, number of removals during data cleaning and final values post cleaning. Data extracted for patients treated with either palliative or curative intent at the RT department at the Sahlgrenska University Hospital in Sweden.

	**Initial raw data No.**	**Duplicates** **No. (%)**	**NULL intent** **No. (%)**	**Final No.**
*Referral data (For all 84 diagnoses)*				
Patients	3916	28 (1)	578 (NULL diagnosis [1 0 3], blank rows [1 0 7], irrelevant entries* [3 6 8]) (15)	**3310**
*Appointment data (For all 84 diagnoses)*				
Mould	1662	36 (2)	66 (4)	1560
CT- Site1	3735	41 (1)	52 (1)	3642
MRI	684	13 (2)	17 (2)	654
QA	1285	304 (24)	35 (3)	946
*Fraction data (For all 84 diagnoses)*				
Fractions	58,228 fractions	None	4 (<1)	58,224

Abbreviations: CT = computed tomography, Initial No. = original number obtained from the raw data before data cleaning, MRI = magnetic resonance imaging, QA = quality assurance.

*An irrelevant entry was defined as the entry with an unrecognized format or entry with corrupted data / meaningless data.

### Substitution strategies for treatment intent

After having removed duplicates and null values, 373/3310 (12 %) of patient referrals had unknown treatment intent entries. Out of the remaining 2937 referrals with known treatment intent, 69 % had curative and 31 % had palliative intent. From the four investigated substitution strategies, the ratio substitution fared best with closest percentages and ordering for the six largest diagnosis-intent groups compared to the reference dataset (Table 3). Furthermore, the remaining diagnosis-intent groups of the reference dataset remained identical for the ratio substitution strategy with small changes in the ordering from groups 7 to 20. The other three strategies resulted in up to three diagnosis-intent groups that were not part of the largest 20 groups of the reference dataset.

**Table 3:** Substitution strategy results in terms of the sequence and percentage distributions of the largest 20 diagnosis and intent groups for all four strategies tested on the automatically-cleaned dataset from 2015 to 16 compared to the manually-cleaned dataset from that same time period.

			Automatically-cleaned dataset							
No.	Manually- cleaned dataset (reference)		All C (Strategy 1)		All P (Strategy 2)		50–50 (Strategy 3)		Ratio (Strategy 4)	
	Diagnosis -Intent	%	Diagnosis -Intent	%	Diagnosis -Intent	%	Diagnosis -Intent	%	Diagnosis -Intent	%
1	C50 - C	22 %	C50 - C	18 %	C79 – P*	*20 %*	C79 – P*	*19 %*	C50 – C	21 %
2	C77 - P	16 %	C79 – P*	*19 %*	C50 – C*	*17 %*	C50 – C*	*16 %*	C77 – P	17 %
3	C61 - C	11 %	C61 - C	10 %	C61 - C	11 %	C61 - C	10 %	C61 - C	9 %
4	C34 - C	6 %	C34 - C	6 %	C34 - C	6 %	C34 - C	6 %	C34 - C	6 %
5	C34 - P	5 %	C34 - P	6 %	C34 - P	5 %	C34 - P	4 %	C34 - P	6 %
6	C71 - P	2 %	C61 – P*	*4 %*	C61 – P*	*5 %*	C61 – P*	*4 %*	C71 - P	3 %
7	C61 - P	2 %	C71 - P	2 %	C71 - P	2 %	C71 - P	2 %	C61 - P	2 %
8	C50 - P	1 %	C54 - C	1 %	C50 - P	1 %	C20 - C	2 %	C90 - P	2 %
9	C90 - P	1 %	C90 - P	1 %	C90 - P	1 %	C50 - P	1 %	C09 - C	1 %
10	C15 - C	1 %	C50 - P	1 %	C54 - C	1 %	C09 - C	1 %	C54 - C	1 %
11	C54 - C	2 %	C09 - C	1 %	C09 - C	1 %	C90 - P	1 %	C53 - C	1 %
12	C09 - C	2 %	C35 - C	1 %	C20 - C	1 %	C54 - C	1 %	C50 - P	1 %
13	C20 - C	2 %	C20 - C	1 %	C35 - C	1 %	C53 - C	1 %	C20 - C	2 %
14	C53 - C	1 %	C43 - C	1 %	C53 - C	1 %	C15 - C	1 %	C15 - C	2 %
15	C01 - C	1 %	C77 - P	1 %	C77 - P	1 %	C77 - P	1 %	C77 - P	1 %
16	C44 - C	1 %	C83 - C	1 %	C01 - C	1 %	C83 - C	1 %	C21 - C	1 %
17	C77 - C	1 %	C44 - C	1 %	C44 - C	1 %	C01 - C	1 %	C01 - C	1 %
18	C83 - C	1 %	C01 - C	1 %	C83 - C	1 %	C44 - C	1 %	C44 - C	1 %
19	C21 - C	1 %	C21 - C	1 %	C21 - C	1 %	C21 - C	1 %	C83 - C	1 %
20	C49 - C	1 %	C79 - C	1 %	C77 - C	1 %	C77 - C	1 %	C49 - C	1 %
Total (20)	2599		2901		2912		2891		2877	
Total patients ALL (84)	3209	**81 %**	3310	78 %	3310	79 %	3310	75 %	3310	**81 %**

Abbreviations: 50–50 = Split substitution between curative and palliative, All C = All curative, All P = All palliative, C-Curative intent, CXX: ICD-code for specific diagnosis, P-Palliative intent, Ratio = Palliative to curative ratio-based substitution per diagnosis, No. = Number indicating ordering of diagnosis and intent groups in the manually-cleaned reference dataset.

*=diagnosis intent category dissimilar to the manually-cleaned dataset for the top six.

Note that the 20 largest diagnosis and intent groups corresponded to 80% of all data.

### Comparisons between the manually cleaned reference data and the automatically cleaned tool data

In comparison to the reference dataset, the overall differences in the total number of referred patients, number of appointments for different tasks and fractions for the tool dataset ranged between −5% to 5 % (Table 4). The smaller differences were found for Mould, TP andQA (<1%), and the larger differences were found for Fractions and MR (±5%). The tool data were numerically overestimated in 80/140 (57 %) of comparisons.

**Table 4:** Referral, appointment and fraction data statistics for the manually cleaned and automatically cleaned datasets from 2015 to 16.

**Diagn-osis**	**Patients**			**Mould**			**CT**			**MR**			**TP**			**QA**			**Fractions**		
**Intent**	REF	DET	DET-REF (%)	REF	DET	DET-REF (%)	REF	DET	DET-REF (%)	REF	DET	DET-REF (%)	REF	DET	DET-REF (%)	REF	DET	DET-REF (%)	REF	DET	DET-REF (%)
**C50 - C**	−716	717	1 (0)	45	47	2 (4)	748	726	−22 (−3)	0	0	0 (N/A)	837	797	−40 (−5)	0	11	11 (N/A)	14,299	13,859	−440 (−3)
**C77-79 - P**	515	504	−11 (−2)	305	308	3 (1)	612	639	27 (4)	33	31	−2 (−6)	673	631	−42 (−6)	36	41	5 (14)	2094	1982	−112 (−5)
**C61 - C**	345	349	4 (1)	1	1	0 (0)	339	318	−21 (−6)	339	307	−32 (−9)	356	328	−28 (−8)	238	211	−27 (−11)	9830	9281	−549 (−6)
**C34 - C**	204	234	30 (15)	109	118	9 (8)	232	227	−5 (−2)	3	9	6 (200)	257	224	−33 (−13)	48	41	−7 (−15)	4111	3962	−149 (−4)
**C34 - P**	162	192	30 (19)	128	136	8 (6)	185	191	6 (3)	3	5	2 (67)	215	204	−11 (−5)	18	16	−2 (−11)	1418	1424	6 (0)
**C71 - P**	68	68	0 (0)	68	65	−3 (−4)	74	67	−7 (−9)	50	46	−4 (−8)	73	65	−8 (−11)	27	31	4 (15)	1275	1178	−97 (−8)
**C61 - P**	56	92	36 (64)	24	27	3 (13)	60	90	30 (50)	9	3	−6 (−67)	71	68	−3 (−4)	8	5	−3 (−38)	370	410	40 (11)
**C50 - P**	41	82	41 (100)	18	26	8 (44)	47	22	−25 (−53)	2	2	0 (0)	49	46	−3 (−6)	1	3	2 (200)	241	244	3 (1)
**C90 - P**	40	66	26 (65)	20	21	1 (5)	47	47	0 (0)	0	3	3 (N/A)	56	54	−2 (−4)	1	6	5 (500)	214	224	10 (5)
**C15 - C**	44	65	21 (48)	42	47	5 (12)	46	49	3 (7)	3	0	−3 (−100)	63	56	−7 (−11)	12	17	5 (42)	1224	1224	0 (0)
**C54 - C**	56	64	8 (14)	11	12	1 (9)	63	68	5 (8)	2	2	0 (0)	60	57	−3 (−5)	48	38	−10 (−21)	1448	1457	9 (1)
**C09 - C**	49	61	12 (24)	50	56	6 (12)	46	47	1 (2)	46	46	0 (0)	54	54	0 (0)	48	41	−7 (−15)	1622	1711	89 (5)
**C20 - C**	49	59	10 (20)	1	1	0 (0)	46	33	−13 (−28)	0	3	3 (N/A)	54	58	4 (7)	29	21	−8 (−28)	821	819	−2 (0)
**C53 - C**	44	54	10 (23)	3	4	1 (33)	38	33	−5 (−13)	6	3	−3 (−50)	60	67	7 (12)	52	43	−9 (−17)	1253	1288	35 (3)
**C01 - C**	38	51	13 (34)	40	38	−2 (−5)	35	9	−26 (−74)	30	26	−4 (−13)	39	34	−5 (−13)	41	32	−9 (−22)	1270	1123	−147 (−12)
**C44 - C**	31	50	19 (61)	31	30	−1 (−3)	32	27	−5 (−16)	2	0	−2 (−100)	36	36	0 (0)	13	9	−4 (−31)	713	782	69 (10)
**C77 - C**	48	49	1 (2)	20	20	0 (0)	4	33	29 (725)	10	26	16 (160)	58	28	−30 (−52)	24	26	2 (8)	837	876	39 (5)
**C83 - C**	32	49	17 (53)	34	31	−3 (−9)	37	58	21 (57)	0	3	3 (N/A)	40	37	−3 (−8)	9	12	3 (33)	506	504	−2 (0)
**C21 - C**	35	45	10 (29)	4	4	0 (0)	9	33	24 (267)	0	0	0 (N/A)	46	41	−5 (−11)	34	32	−2 (−6)	935	911	−24 (−3)
**C49 - C**	26	26	0 (0)	26	19	−7 (−27)	28	29	1 (4)	0	0	0 (N/A)	29	28	−1 (−3)	3	1	−2 (−67)	556	598	42 (8)
**TOTAL (20)**	2599	2877	278 (11)	980	1011	31 (3)	2728	2746	18 (1)	538	515	−23 (−4)	3126	2913	−213 (−7)	690	637	−53 (−8)	45,037	43,857	−1180 (−3)
**Other**	610	433	−177 (−30)	573	548	−25 (−4)	834	896	62 (7)	149	135	−14 (9)	795	1003	214 (27)	258	309	51 (20)	10,497	14,367	3870 (37)
**MEDIAN diff.**	**22 %**			**6 %**			**9 %**			**32 %**			**21 %**			**5 %**			**7 %**		
TOTAL ALL	3209	3310	101 (3)	1553	1559	6 (0)	3562	3642	80 (2)	687	650	−37 (−5)	3921	3916	−5 (0)	948	946	−2 (0)	55,534	58,224	2690 (5)

Note that the third column under every sub-table (DET-REF[%]) indicates the difference in manually cleaned and automatically cleaned data represented in values and in percentages.

Abbreviations: C = curative intent, CT = computed tomography, CXX = ICD-codes for specific diagnosis, **DET = data extraction tool/automatically cleaned tool dataset**, Median Difference = median difference for largest 20 diagnosis = intent categories, MR = magnetic resonance (imaging), P = palliative intent, QA = quality assurance, **REF = reference/manually-cleaned dataset**, TP = treatment plans. TOTAL (20) = total for the largest 20 diagnosis groups. Other = combined total for the remaining diagnosis groups. Median difference = median difference in the values from the largest 20 diagnosis groups.

Note that the 20 largest diagnosis and intent groups corresponded to 80% of all data.

### External application example – Using both manually- and automatically-cleaned datasets from two time periods as input data

**Fig. 3:**
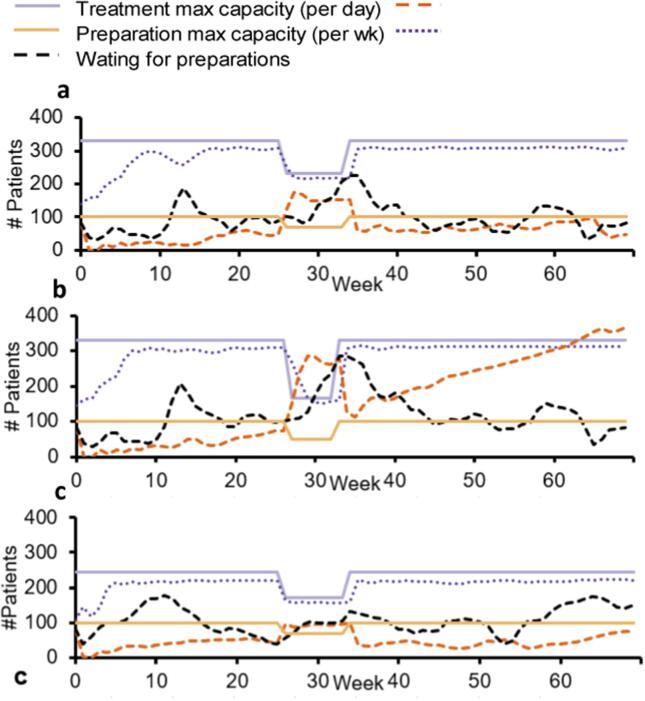
Simulation results for radiotherapy preparation and treatment steps during an eight-week summer vacation period for the radiotherapy department at the Sahlgrenska University Hospital in Sweden. a. manually-cleaned reference dataset for 2015–16, b. automatically-cleaned tool dataset for 2015–16, and c. automatically-cleaned tool dataset for 2020–21. The data here represents patients from the largest 20 diagnosis and intent groups corresponding to 80% of all patient data.

**Fig. 4:**
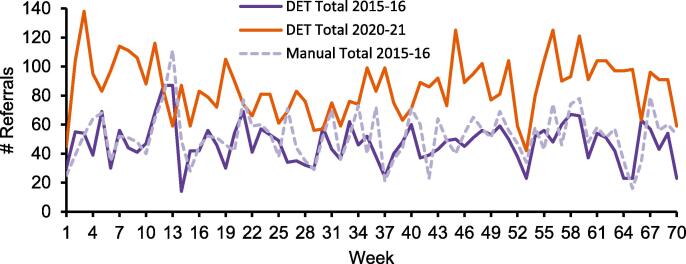
Yearly referral inflow pattern at the radiotherapy department at the Sahlgrenska University Hospital in Sweden for the investigated manually-cleaned and automatically-cleaned patient datasets. The data here represents all patient data from 2015 to 16 and 2020–21 with 84 different diagnoses. Abbreviation: DET = Data Extraction Tool.

Simulation results for the example external application based on the manually-cleaned reference dataset and the automatically-cleaned tool datasets for the time periods 2015–16 and 2020–21 are shown in Fig. 3 and Fig. 4. The largest 20 diagnosis and intent groups corresponded to 80 % of all data.

The outputs from the 2015–16 datasets differed with respect to the pattern of patients waiting for treatment in the automatically cleaned data compared to the manually cleaned reference data. The patterns of patients waiting for preparation and patients under treatment were otherwise similar between the two datasets.

Over time, the ratio of curative to palliative treatments had remained similar but referral, appointment and fraction data had increased in 2020–21 in comparison to the situation in 2015–16. There were 81 % more referrals, 31 % more appointments, and 21 % more fractions. The output from the simulation model showed a somewhat different pattern of patients waiting for preparations in 2020–21 than in 2015–16. However, the pattern of patients waiting for treatment and patients under treatment showed uniform behaviour for both time periods in relation to their respective preparation and treatment capacities.

## Discussion

In this work, we explored an automated data preparation approach based on previously used manual data cleaning and formatting principles for OIS data in RT. We successfully created and verified the ability of a novel data-extraction tool to time-efficiently prepare ready-to-use datasets for an external application of the RT domain. Using real patient data from a large modern RT department in Sweden, we found that original OIS data included both duplicated and missing information motivating both removal and substitution strategies in the data cleaning process. Information relating to referrals generally included more ambiguities than information relating to appointments for different RT subtasks including treatment fractions. Using a ratio substitution strategy for missing information on treatment intent resulted in numerically overall small differences between the investigated manually-cleaned reference dataset and the automatically-cleaned tool dataset for the same time period. The identified differences did not affect the output from the investigated external application.

Radiation oncology is one of the most quantitative disciplines in the medical field, but tools which effectively make use of clinically registered data to support decision making in different RT settings are lacking [9]. A PubMed search on July 25th, 2022, gave only five relevant hits on different combinations of “tools”, “automation”, “extraction”, “datasets” and “radiotherapy”. All five publications were based on automatic extraction of RT data but used purpose-specific extraction features, and only supported explicit database structures. Two of these publications related to tools that were specifically or partly developed to capture dose/volume statistics for RT studies with researchers reporting the dataset preparation to be both labour intensive and challenging [6], [7]. In the study by Gong *et al.* in 2016, they compared the time to use their developed tool to automatically extract MIMvista data for dosimetry review according to a specific trial protocol with the time needed to obtain the same information manually [6]. They found that the automatic extraction for a small-scale example (dose/volume points for 20 dose-volume histograms) was completed in 3 min whilst the manual work took 1 h. In the other more recent study by Stervik *et al.*, they analyzed radiation-induced toxicity for lung cancer treatments with data collected from multiple hospitals. They addressed both the time aspect and challenges with RT data inconsistencies since the databases in question were not entirely consistent in terms of the availability and coordination between patient and treatment related data. They report that analysis of the extracted data with inconsistencies would have resulted in an unfitting selection of patient profiles for their study and especially found the identification of complex inconsistencies to be a lengthy task. They also noted that there are no strict guidelines for linking unique patient data to the corresponding appointment data in RT. In our experience, manual processing of such raw (atomic) data can initially take up to several weeks (primary preparation) while dataset updates take somewhat less (secondary preparation). Our tool overcomes these limitations by introducing a strategy which automates all the laborious procedures required to clean and process patient and appointment data. Its GUI is designed to be user friendly for the hospital staff. The execution to export the here investigated formatted datasets for both primary and secondary preparations took < 15 s.

To generate a consistent dataset for external use from the raw dataset in our study, a substantial number of NULL and duplicate entries in both patient and appointment data had to be handled. These extracted data were entirely based on the information entered in the OIS-ARIA. Some missing data can potentially be retracted from the Electronic Medical Records (EMR) and manual efforts for retraction should be taken into consideration before applying any dataset-manipulation strategy [9]. However, If the data cannot be retraced, a substitution strategy for missing information can improve overall data veracity. Our dataset from 2020 to 21 included a high number of referrals with unknown treatment intents, which made a substitution strategy critical. Close to 40 % of data would have been lost if all missing intent referrals had been removed. The 2015–16 dataset had lower number of missing information so a substitution strategy was less critical for this dataset. Another strategy to handle missing data was implemented by Beesley*. et. al.* in 2019 where they used Markov chain Monte-Carlo algorithm to avoid the bias arising from missing OIS/RT data [10]. This strategy represents the use of probabilistic inference to stabilize the dataset pattern by only keeping the patient entries with no missing information. This did not affect their overall analysis since their study used more than 17 years of patient data. Such large time periods may not be available for some external applications.. To handle various dataset sizes, our strategy focuses on a pre-removal solution to retain the veracity by lowering the removal rate with probabilistic substitution first. In addition to challenges in handling missing data from the OIS database, patterns in RT may also change over time. Patient referral volumes are naturally affected due to the worldwide increasing number of cancer patients [11]. Along with that, appointment/fraction data may differ periodically given new evidence on treatment strategies or resizing of an RT department. For example, a recent 5-year trial including 4096 breast cancer patients, showed that a short-course radiation regimen (26 Gy; 5 fractions; 1 week) was as safe and effective as a protracted treatment course (40 Gy; 15 fractions; 3 weeks) [12]. If accepted in the clinic, such results dramatically change the overall throughput of patients and the associated digital RT landscape. In our dataset from 2020 to 21, we noted a peculiarly high surge for curative prostate cancer patients (C61-C) with 279 % more referrals than in the dataset from 2015 to 16. The primary reason for this was the incorporation of a 3-linac satellite department’s data into the same OIS. To adapt to the abovementioned and other non-linear changes at RT departments, our tool by design supports external applications with yearly statistics for the specified variables.

Strengths of our study include the use of real data from a large RT department to develop, test and verify the tool, including extensive datasets from two separate time periods. Our developed tool can be used by other RT departments with minor changes in the database queries. It can be customized for other external applications in clinical or non-clinical settings. For example, the tool can present statistics on historical data (summarized data with indexes like mean, median and standard deviation), and it can support time-sensitive applications with real-time cleaned datasets. Currently, the data extraction feature is only available for the ARIA (Varian) OIS. For non-Varian platforms, the tool can still be used for cleaning and preparation but requires that the extracted data from non-Varian databases are specified on a pre-defined input format. One limitation with our strategy is the substitution algorithm, which was based on the available data in the selected time frame. If available data are small in numbers, the substitution ratios can be biased and may influence the overall output from the external application. This can be handled by basing the substitution algorithm on historical data, if available, rather than just the selected time frame to improve data veracity. To simplify the patient-appointment data linking process, we assumed that the yearly number of patients and respective appointments were fitted within the investigated time frame. However, in reality, patients may have a larger gap (2 months or more) between referral and scheduled pre-treatment tasks having appointments falling outside a selected time frame. This can introduce a slight skewness in the distribution of appointments, but for larger datasets, as investigated in our study, this will typically be balanced by the set of appointments for such patients from an older time frame. In case of smaller datasets, the tool itself is equipped to handle these data ambiguity situations. When it comes to institute-specific issues which can introduce systematic errors, additional procedures or configuration changes can be implemented in the tool to compensate for this given that the concerns are brought forward and can be quantified. To summarize, our in-depth data cleaning and preparatory algorithm provides a reliable and fast approach to produce datasets for external use and is available on request.

In conclusion, preparing OIS data for external applications can be time consuming. We successfully implemented a software tool to prepare ready-to-use OIS datasets for external applications. The evaluations for our investigated application showed overall results close to the manually-prepared dataset. Our tool can import data from a specified OIS and will automatically clean and prepare the information before formatting it for external use. In our experience, the time taken to prepare the dataset using our automated strategy can reduce the time for manual preparation from weeks to seconds. This novel approach can help to efficiently manage OIS data for external use and can bolster continuous data-driven development in RT departments.

## Funding

This work was supported by The Swedish Research Council under Grant 2017-01735, the Swedish state under the agreement between the Swedish government and the county councils, the ALF-agreement under Grants ALFGBG-72011 and ALFGBG-965800, the Kamprad Family Foundation for Entrepreneurship, Research and Charity under Grant 20190083, and Jubileumsklinikens Cancer Research Foundation 2021:347.

## Declaration of Competing Interest

The authors declare that they have no known competing financial interests or personal relationships that could have appeared to influence the work reported in this paper.
