# Supplementary material for: Automated data extraction tool (DET) for external applications in radiotherapy
Source: Tech Innov Patient Support Radiat Oncol. 2022 Dec 20;25:100194. doi: 10.1016/j.tipsro.2022.12.001 (PMC9842687; doi:10.1016/j.tipsro.2022.12.001)
Supplement: Supplementary Data 1 [file mmc1.docx]

# Supplementary Material

## Data cleaning steps

To assure consistency in the manual data cleaning process, written guidelines (in Swedish) were created and explicitly followed by one project assistant under the supervision of a senior medical physicist (J.L.). The guidelines included instructions for sorting/filtering procedures and steps for verification.

### Removing inconsistencies

For patient referrals, there were numerous NULL entries for diagnosis with a NULL value indicating missing information for type of diagnosis. There were also duplicated patient appointment identifiers (ids) indicating two or more appointments (mould/imaging/QA) with different diagnoses for a same patient referral. For such duplicate entries, we compared referral dates with scheduled start dates and retained the entry closest to the scheduled start date. Occasionally, merging information from different data tables in the OIS database or missing information from the database generates duplicated information. A patient with two or more treatment paths or with a deliberately altered treatment path, e.g., when redoing imaging after a certain number of fractions, was not considered as a duplicate if unique appointment ids for each path could be identified. Both NULL entries and duplicated information were automatically removed using so-called events in C# (details below).

The logic behind removal of both NULL values and duplicated information was to make the data consistent with every patient referral having a single entry for all unique tasks throughout the RT workflow per prescribed treatment. To achieve this, we kept the original entry and removed the duplicates by recognizing them with their NULL timestamps.

### Substitution strategies

For treatment intents, there were multiple ‘unknown’ entries in both referral and appointment data with ‘unknown’ indicating missing information. For each diagnosis group, this missing information was assigned either curative or palliative intent using four substitution strategies where the missing information was replaced using: 1) 100% curative intent, 2) 100% palliative intent, 3) 50% split between curative and palliative, and 4) rate of curative/palliative based on the ratio of known curative to known palliative referrals for each diagnosis. The best-balanced substitution strategy for each diagnosis group was identified by assessing the ratio of each strategy in comparison to the reference dataset ratio for the group in question.

## Tool development

The tool for automatic data extraction was primarily built in C# (Visual Studio IDE, Version 17.1, Microsoft, Washington, U.S.A) with Excel automation queries. It was created to enable seamless extraction, cleaning and formatting of data when triggered by the user to update information for applications with a pre-determined input format. For the example external application tested here, the investigated input data format required the structuring of data according to the largest diagnosis and treatment intent groups which included approximately 80% of the total number of patient referrals and associated statistics for usage of tasks/resources in the RT workflow. The remaining smaller diagnosis-intent groups were merged into a ‘other’ group.

The tool was also required to support data formats for scenarios where patient diagnosis was used without treatment intent. Both formats (with and without intent) were possible in the external application in question. Refer to figure 1 for the overall process and table 1 for the complete procedure.

### C# automation

C# automation includes linking and automating data extraction and data cleaning. The extraction was implemented in C# and executed through C# - OIS server database connectivity.

The algorithm for data cleaning was implemented in C# and executed through the windows application .NET framework. An event in C# consolidates various calculations on the back-end (where algorithmic steps are executed) into single click operations on the front-end (where the user interacts with the program through menus or buttons/icons). The cleaning and formatting steps described above take place on the back-end but are triggered by the user via the tool’s graphical user interface (GUI; figure 2). Cleaned and formatted datasets are then automatically exported to a user-defined location on the computer connected to the OIS database server in a few seconds. Data cleaning steps can be adjusted according to the requirement of other external applications by changing dataset variables, user configurations and adding calculations to the back-end. To allow flexibility in selection of time periods from which data are to be extracted, the GUI also includes a date-time picker.

### Excel automation

Pre-set formulae were written in Excel to calculate percentage of appointments, number of fractions per diagnosis and number of referrals for a specific diagnosis. This calculation file was linked to the extracted and cleaned dataset file through C#.

Every time a new dataset is to be extracted, the calculation file is automatically updated with results based on the new dataset and is then ready to be imported into the external application. The same process can take place in a loop where extraction and updating of linked calculation results can be scheduled to perform automatically within any specific time interval.

### Accessing the tool

The tool can be easily accessed on any computer system compatible with Microsoft visual studio and which is connected to the department’s OIS server. The tool acts as a regular windows application and one can click on the executable file of the tool to open the GUI and start the application.
